# Molecular Docking of Phytoligands to the viral protein receptor P. monodon Rab7

**DOI:** 10.6026/97320630013116

**Published:** 2017-04-30

**Authors:** Jerrine Joseph, Raj Bhaskaran, Muthusamy Kaliraj, Muthiyah Muthuswamy, Arumugam Suresh

**Affiliations:** 1 Centre for Drug Discovery and Development, Sathyabama University, Jeppiaar Nagar, Rajiv Gandhi Salai, Chennai-600 119, Tamil Nadu, INDIA;; 2 Research and Development Centre, Sathyabama University, Jeppiaar Nagar, Rajiv Gandhi Salai, Chennai-600 119, Tamil Nadu, INDIA;; 3 Thiruvalluvar University Constituent College of Arts and Science, Kallakurichi-606204, Tamil Nadu, INDIA;

**Keywords:** Drug target, WSSV, P. monodon Rab7, Homology modeling, Phytoligands

## Abstract

The development of shrimp aquaculture has been severely affected by viral diseases resulting in a huge economic burden to the industry. White spot disease (WSD) has caused severe mortality in farmed shrimp in many countries. Globally aquaculture industries face huge economic losses due to rapid spread of White Spot Syndrome Virus (WSSV) disease that can cause 100% mortality in a short period of 3-10 days. In the present study in order to prevent the spread of WSSV disease in shrimps, the receptor, PmRab7 has been chosen as the drug target. Due to the absence of a precise 3D structure of the target, homology-modeling approach was employed to obtain the structure that was validated later. This structure was then used as a template to screen selective phytomolecules as potential antiviral agents and their docking results with the target are analyzed based on their energy scores. Identification of the drug-like molecule obtained from the docking analysis would be used to optimize to a candidate drug. This is expected to play a role of the inhibitor that blocks the binding of the viral protein to the receptor, duly preventing the WSSV disease.

## Background

Viral diseases strongly affect the shrimp aquaculture industry resulting in a heavy loss to the economy, of all the known viruses in the field of aquaculture, such as, Yellow head virus (YHV), Infectious myonecrosis virus (IMNV), Taura syndrome virus (TSV), White spot syndrome virus (WSSV), and Infectious hypodermal and hematopoietic necrosis virus (IHHNV). WSSV exists to be the most devastating shrimp pathogen in cultured shrimp, globally [1-3]. The White spot disease (WSD), resulting from the infection of WSSV has caused severe mortality in farmed shrimps in many countries [4-6]. There is no treatment currently available to control the spread of the disease due to lack of effective therapeutics.

The WSSV genome has been completely sequenced for four isolates, and more than 50 virus-encoded proteins have been identified as structural proteins [7]. Among these structural proteins, the envelope proteins are extremely important because they are believed to be the first molecules to interact with the host and, consequently, play critical roles in cell targeting as well as in triggering host defences. The precise mechanism of entry of WSSV into P. monodon is yet unknown and it is speculated that some receptor proteins viz. viral attachment protein,PmCPB, β – integrin and PmRab7 are involved in directing WSSV into the host. The viral envelope and its structural proteins form the first and most important component of virus to directly come into contact with the shrimp [8,9].

**Figure 1:**
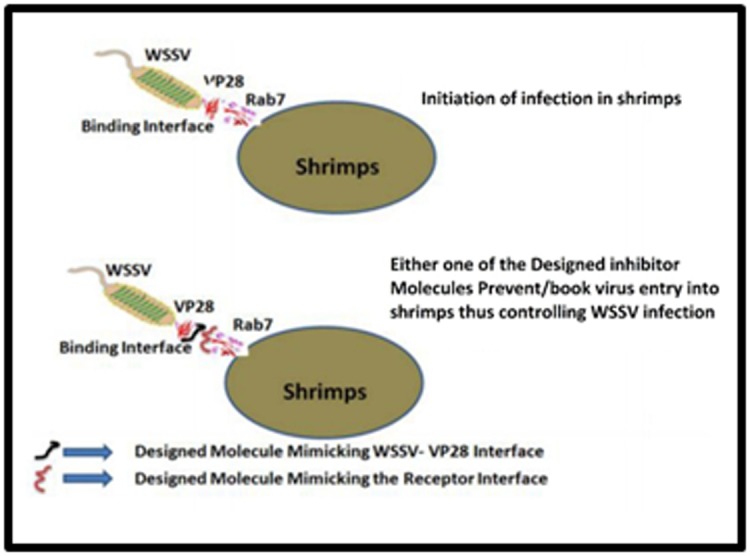
The scheme represents the design of inhibitors for prevention of WSSV Entry into the shrimps. Both possibilities of designing the inhibitors using the viral protein or the receptor respectively have been indicated, but action of only one of the designed molecules complete the task of inhibition.

The virus entry into the host cells occurs through the interactions of viral envelope proteins with the host cellular receptors. These interactions are so specific that inhibitors that could prevent these interactions show promising anti-viral activity. In case of WSSV, VP28 is one of the major envelope proteins that are identified as the interacting partner with the host surface cellular protein P. monodon Rab7 [8,10]. We have been working on an aim at studying the structural details on the residue specific interactions between the WSSV VP28 - Shrimp PmRab7 proteins by employing a wide range of biophysical, especially NMR, and biochemical methods. The outcome of this study identifies the binding interface and specific residues involved in the viral-host protein-protein interactions [11]. This will help us to design new drug like molecules mimicking the envelope protein binding interface or the receptor protein binding groove, such that the molecules that we design from the knowledge of the atomic structure of VP28 / PmRab7 prevent and block the viral entry onto the shrimp host, thus controlling the WSSV infection. The scheme representing the design of inhibitors for prevention of WSSV entry into the shrimps is shown in Figure 1. Further, the high-resolution structural characterization of the protein – protein interfaces deliver us the essential knowledge to design inhibitors with high accuracy.

In line with the above hypothesis, here in the present study, in order to prevent the spread of WSSV disease in shrimps, the receptor, PmRab7 has been chosen as the drug target. The viral protein VP28 could also be considered as another target, as the reverse approach through which the viral infection can be combated too. The use of the designed / synthesized molecule in the experiments to be done in ponds would be difficult and is also not an eco friendly approach. Hence, use of recombinant pmRab7 is considered to be the ideal choice. But the three dimensional atomic structure of the target protein PmRab7 is unavailable in Protein Data Bank. Hence, its 3D model has been constructed using homology-modeling approach.

Plant-derived natural products play a significant role by being a lead molecule in the development of drug candidates. Herbal extracts represent the primary form of health care for a major proportion of the world population and are the important sources of single-molecule drug leads [12]. A prominent example is the anti-malarial activity of Artimisiaannua discovered by Professor Tu, of China Academy of Chinese Traditional Medicine, recipient of the 2015 Nobel Prize for Physiology and Medicine [13,14].

Phytomolecules like Baicalein, Luteolin, Quercetin and Kaempferol are potential antiviral agents against a wide range of important viruses including Dengue, HIV, H5N1 influenza A viruses, Coxsackie virus, CHIKV and Japanese encephalitis virus [15]. The quest for identification of the leads for various drug targets led us to investigate the phytomolecules as antiWSSV leads and the above flavonoids were considered for our present study.

The contribution by homology modeling and computational biology has accelerated the pace of drug discovery. It is used in the biopharmaceutical industry to discover and develop new lead compounds. By this route, one can visualize the possibilities of binding of potential small molecules as ligands / inhibitors. The analyses of the different docked conformations are carried out using the scores / energies based on their binding affinities, as parameters to evaluate the ideal ligand. Therefore, we have designed the present study using computational approaches to discover the potential of these flavonoids targeting PmRab7 and preventing the complex formation as depicted in Figure 1. Though Figure 1 is displayed with two ligands, each one encompassing the interface region of VP28 and Rab7 respectively, the idea is to deal with them independently. In the present study, we concentrate on the surface of Rab7 interacting with VP28. Essentially, we have to design a small molecule that is mimicking this interface so that it can act as a good inhibitor.

## Methodology

### Protein Preparation and Homology Modeling

In the isolation and characterization of white spot syndrome virus (WSSV)-binding proteins from shrimp, out of the three membrane-associated molecules identified, a 25-kDa protein was identified that had a primary structure with high homology to the small GTP-binding protein Rab7, named it Penaeus monodon Rab7 (PmRab7). This is the first Rab homologue from crustaceans to be identified and characterized. It is shown that the GTP-binding protein Rab7 may be a receptor for VP28 envelope protein of WSSV in shrimp. Rab proteins are known to be regulators of vesicle budding and fusion events and represent a family of over 30 proteins that are localized on the surfaces of distinct membrane-enclosed compartments of exocytic and endocytic pathways. They are found in all eukaryotes, including yeasts, plants, insects, and mammals. PmRab7 has the four conserved GTP-binding regions of the small G protein superfamily (G1 and G3 to G5), as well as an effector site (G2). These five regions are characteristic of Rab proteins. The sequence analysis suggests that PmRab7 may be an active GTPase that could cycle between the GDP- and GTP-bound states.

The 3D structure of the PmRab7 protein is unavailable in protein data bank. The protein sequence of Penaeusmonodon Rab7 (Accession No.ABB70064.1) was taken from National Centre for Biotechnology Information (NCBI). The protein sequence was used to develop homology-modeled structure. The threedimensional coordinates of PmRab7 were constructed using Schrödinger [16], a comparative protein-modeling program. It computes a model based on the alignment of the sequence to be modeled with known related 3D structures. The homology modelled 3D structure of PmRab7 in its complex form with the ligand GDP has been obtained from Schrodinger and the model has structural similarity with the rat Rab7. Water molecules, ligands and other heteroatoms were removed from the model. Hydrogen atoms were added to the model using CHARMm force field. Energy minimization was performed by using conjugate gradient method with an RMS gradient of 0.01kcal/Å mol on Accelyrs-Discovery studio client [17].

### Model Validation and Structural Characterization

Structure verification programs such as PROCHECK, Ramachandran Plot and VERIFY3D were used from the Structure Analysis and Verification Server (http://nihserver.mbi.ucla.edu/SAVES/) to evaluate the validity of the homology modeled PmRab7. These programs validate the homology-modeled structure by checking the quality of the modeled structure, its resolution and refinement. PROCHECK, the structure verification program checks the stereo-chemical quality of the protein structure and determines the quality of the predicted structure by assessing parameters such as bond lengths and angles, planarity of the peptide bonds, geometry of the hydrogen bonds, and side chain conformations of protein structures as a function of atomic resolution. Ramachandran Plot visualizes energetically allowed regions for backbone dihedral angles ψ against φ of amino acid residues in protein structure. The Verify3D assess protein structures using three-dimensional profiles. It analyzes the compatibility of an atomic model (3D) with its own amino acid sequence (1D) by assigning a structural class based on its location and environment (alpha, beta, loop, polar, apolar etc). Then a score for each of the 20 amino acids in this structural class is obtained from a database generated from good structures. The scores range from -1 (bad score) to +1 (good score).

### Ligand Preparation

Among the plant-derived flavonoids, the potential phytochemical ligands namely Kaempferol ,Luteolin, Baicalein, and Quercetin were chosen based on the literature survey as potent compounds possessing antiviral activity. The molecules were docked against PmRab7. Their basic structure is a skeleton of diphenylpropane, namely, two benzene rings (two extreme rings) linked by a threecarbon chain that forms a closed pyran ring (heterocyclic ring containing oxygen, the middle ring) with benzenic first ring. These phytochemicals were retrieved from the pubchem database and the chemical structures were generated using SMILES (Simplified Molecular Input Line Entry Specification) by using the Discovery Studio 2.4 version. Also considered in this analysis is GDP (guanosinediphosphate), the ligand that is being complexed in the crystal structure of rat Rab7. GDP consists of three moieties: The guanine ring, which is a nitrogen-containing heterocycle; the ribose sugar ring; and the diphosphate group. The structural details namely, the chemical name, PubChem ID and Molecular formula of the selected phytochemicals along with GDP are given in Table 1.

### Drug likeliness prediction

Drug like properties of the ligands were predicted by using the Discovery Studio 2.4 version. The Lipinski’s rule helps in distinguishing drug-like and nondrug-like properties and predicts high probability of success or failure due to drug likeliness of the molecules. The Lipsinki’s filter helps in early preclinical assessment, thereby avoiding costly late stage preclinical and clinical failures. Lipinski’s rules state that ideal drug molecules possess 5 hydrogen bond donors, not more than 10 hydrogen bond acceptors, Molecular weight not more than 500 and LogP not more than 5 [18].

### Molecular Docking

The grid-based molecular docking method is used here using the program Cdocker (Accelrys) that employs CHARMmforce field. The targetis held rigid while the ligands are allowed to be flexible during the refinement. Since the ligand, GDP was already complexed in the crystal structure; the same binding site info is considered. Hence, it is possible, however, to specify the ligand placement in the active site using a binding site sphere. For this purpose, the GDP ligand present in the active site of the target protein was used to generate the sphere around the active site. Then the prepared ligands are docked to the active site using default parameters. The results of the docking enabled the ranking of the docked conformation of the ligands according to their Cdocker energy values.

### Analyses of the ligand binding sites

The docking poses were ranked according to their docking energies. The scoring function in Cdockerwas used to predict the binding affinity of one ligand to the target molecule. In addition to the structural information, each record includes the Cdocker energy reported as negative value, where the higher value indicates a more favorable binding. This enables the energy to be used like a score. This score includes internal ligand strain energy and receptor-ligand interaction energy, and is used to sort the poses of each input ligand. The molecular visualizations of the docked complexes were analyzedusing the Discovery Studio 2.4 version [17].

## Results

**Figure 2:**
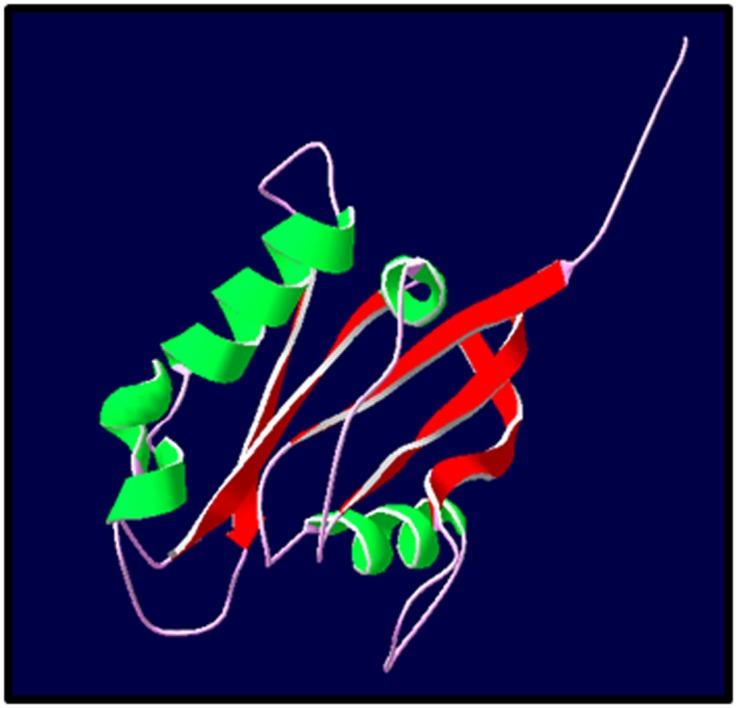
Ribbon diagram of the homology modeled PmRab7. This model resembles the rat Rab7. The model consists of five helices, a double, and triple stranded beta sheets, globular in conformation with the turns protruding outside the globularity.

In this study, theshrimp receptor protein, Pm Rab7, was considered as the target protein towards the lead identification. There are no 3D atomic structures available for the target. Hence, the aim of this study consisted of two parts. First is to build the homology model of the receptor PmRab7 and then to validate and characterize the constructed model. Secondly, use the constructed model as the target to dock with the selected Phytoligands. The homology model was constructed and the 3D structure of shrimp receptor protein PmRab7 was designed by using molecular modeling software Schrödinger. The coordinates of the resultant model were used as the template for the target. The ribbon diagram of the homology modeled PmRab7 is as shown in Figure 2.

As the model has been derived from the structural homology principles, based mainly on Rat Rab7of RattusNorvegicus, the resultant modeled protein has been found to be complexed with the ligand GDP and has been considered for the docking study. Rab7 is a small GTPase and act like a switch which is turned on and off by GTP and GDP molecules. The active site that has been occupied by the ligand GDP was used for the docking of the phytochemical ligands. Four phytochemical ligands belonging to flavonoids class of compounds were considered as the ligands for this computational study. The drug likeliness of the selected ligands along with that of GDP has been presented in Table 2. The table provides the details of the molecular weight, number of donor and acceptor atoms and AlogP values. The data would reveal the likelihood of the ligands that could form a potential lead for further analysis.

Table 3 lists the results of the docking analysis by providing the Cdocker energy for all the selected ligands with PmRab7 as the target. Higher the negative energy corresponds to the stable binding with the target. The Cdocker energy for GDP was also considered and it is proved to be a better complex with PmRab7 [10]. The comparison of the energies of the selected ligands with that of GDP gives an indication about the strength of the ligand binding with the target.

**Figure 3:**
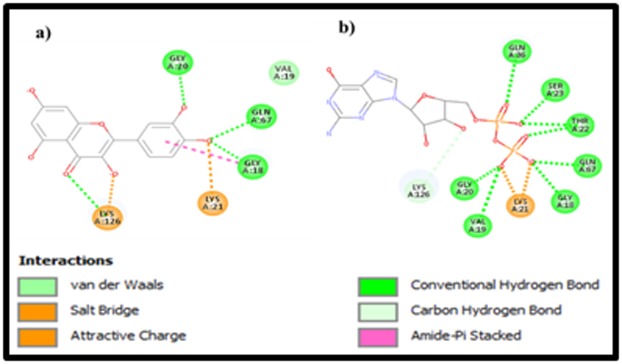
The 2D representations of the intermolecular interactions of the target PmRab7 residue with the ligands (a) Quercetin, (b) GDP

From the docked poses of the ligands with PmRab7, one gets a clear picture about the binding of the ligand with the target. The stability of the docking is decided based on the number of hydrogen bonds / Charge Interactions / Hydrophobic bonds formed. The total Interaction bond counts and the specific residues involved in the interactions of GDP and Quercetin with PmRab7are given in Table 4. Further, the intermolecular interactions between these ligands with PmRab7 have been shown as a 2D representation in Figure 3 (a) for GDP, (b) for Quercetin.

## Discussion

With the non-availability of the atomic structure for the target Pm Rab 7, a homology model was constructed. This model resembles the rat Rab7 with the exclusion of about 40 residues at the Cterminus. The model consists of five helices, a double, and a triple stranded beta sheets (Figure 2). It is globular in conformation with the turns protruding outside the globularity. The structure Analysis and Verification Server have validated the success of the constructed model, which consist of the individual programs such as PROCHECK, Ramachandran Plot and VERIFY3D. According to PROCHECK, that is based on the stereochemical quality, the structure is refined to a 1.5 A resolution. The Ramachandran plot and the plot statistics confirm the good quality of the structure. Over all, the model has good quality with more than 96.8 % of residues under the most favoured and additional allowed regions in the Ramachandran Map. Only two residues are in the disallowed region. Hence, the model is considered as the best with the dihedral angles of the maximum number of residues in the core region and a minimum most in the disallowed region of the Ramachandran Map. From Verify3D, it is observed that nearly 92% of the residues from the homology model of PmRab7 are compatible with its own amino acid sequence, indicating the high quality of the model.

The results of this docking study with the good quality homology modeled Pm Rab7 were analyzed through four different routes, namely, structural visualization and comparison with GDP, the drug like property analysis, analysis of Cdocker energy and finally, the hydrogen bonding and other ligand interactions with PmRab7.

Simple visualization of the ligand structures given in Table 1 indicates clearly that all the ligands belong to a single cluster and have structural similarity among themselves havingbenzo-γ- pyrone derivatives with phenolic and pyrane rings. The variation in the molecular weights for the ligands is by a mere oxygen and one could expect a consistency of the derivatives that could bind with PmRab7.

The analyses of the Drug Likeliness of the selected phytochemicals indicate that all the ligands satisfy completely the Lipinski’s rules (Table 2) and found as potential candidates to be the leads [16]. Surprisingly, the ligand GDP doesn’t satisfy the Lipinski’s rules of acceptor and donor atoms. Though GDP fits nicely into the catalytic site of PmRab7, it could not be a proper candidate towards the making of a drug. Thus, all the ligands chosen for this study are suitable to be the leads that have the potential to form the drug.

The analyses of the Cdocker energies of the ligands obtained by interacting with PmRab7 clearly support the observation of the single cluster made out of the structural analysis. The ligands Quercetin, Luteolin, Kaempferol and Baicalein show Cdocker energies in the range of -51 to -44 kcal/mol (Table 3). Comparing these with the Cdocker energy value of -59.57 kcal/mol for GDP, the ligand are expected to behave like GDP in binding with PmRab7. In fact, their binding strength would be in the `following order Quercetin, Luteolin, Kaempferol and Baicalein with the values of Cdocker energies -51.87; -48.76; -46.41 and - 44.05 kcal/mol. respectively. Overall, the docking study suggests that Quercetin interacted with the target PmRab7 in a fashion similar to the X-ray studies of the ligand GDP. Since all the four ligands are showing a higher negative value of Cdocker energy comparable to GDP and are having the closer molecular formula, they are the likely candidates to be selected for further studies.

Though all four phytoligands were similar in structure visualization, drug likeliness and high negative value of Cdocker energy, we have considered Quercetin only for analysing the intermolecular interactions between Quercetin and PmRab7, due to the reason that Quercetin is found to be the best among all in all respects. In support of the previous analyses and results (Figure 3 and Table 4), Quercetin interacted with PmRab7 by making four H-bonds among them. These H-bond interactions with Gly18, Gly20, Gln67 & Lys126 were also present between GDP and PmRab7. We were interested to find out the common motifs / residues in PmRab7 that interact with all the ligands. GDP is found to interact with 9 residues (Gly18, Val19, Gly20, Lys 21, Thr 22, Ser 23, Gln 36, Gln67& Lys126) of PmRab7. Quercetin has all four residues in common in GDP. The superiority of Quercetin is observed from the hydrogen bonds formed with the residues of PmRab7 to its core ring structures, whereas in GDP, the hydrogen bonds are only to the extended diphosphate region. More hydrogen bonding cause the lowest Cdocker energy in GDP, but with no interaction with the core ring group, it may not be the proper lead molecule. Thus, this analysis strongly supports the earlier observation that Quercetin is best among the four and might act as the potential inhibitor against WSSV. This would be verified and validated by conducting “wet lab” experiments and can be taken up further for confirming as a potential drug candidate.

In order to validate our claim, we are working on the following, as part of our wet lab studies based on our new proposal on the protein-protein interactions between VP28 and PmRab7. We are independently working on the expression and purification of these proteins by recombinant methods. These proteins would be labelled with N15 and C13 to have samples for Multidimensional NMR studies. We also plan to demonstrate the protein crystallization of Rab7 for the X-ray analysis and then use this model for searching potential inhibitors. Protein complex crystallization experiments would also be attempted by our structural biology collaborators to determine the VP28 and PmRab7 complex structure. Since the model showed that Quercetin was very likely to interact with PmRab7, we plan to validate the interaction easily using the following techniques, isothermal titration calorimetry and surface plasmon resonance. We also plan to test if the interaction between VP28 and PmRab7 is disrupted by adding of Quercetin in vitro, following the GST pull-down assay conditions provided by Sritunyalucksana et al.2006 [9]. We could also conduct some WSSV challenge tests with potential inhibitors, to test whether Quercetin can reduce the mortality caused by WSSV.

## Conclusion

The White Spot Disease occurring due to WSSV is a major threat to shrimp farming as it can cause complete mortality of the infected shrimps within few days. There is an urgent need to find effective inexpensive anti-WSSV drugs from natural sources to prevent the infection. The target for this structural and computational host pathogen interaction studies is considered as the shrimp receptor Pm Rab7. But, the major hurdle in having an ideally usable target for anti WSSV drug is the 3D structure of PmRab7.Hence, homology modeling was used to build a 3D structure of PmRab7 and it was successfully validated by the standard procedures.

Plants have derived effective antiviral drugs for some of the viruses of both plant and animal origin. Working along this direction, the present research work is based on the screening of the Phytochemicals that could act as the leads towards the development of the drugs. Hence docking studies were performed that depicted more affinity of binding of Quercetin with PmRab7. Out of the Phytochemicals that were screened, Quercetin is predicted to be the suitable lead to proceed further. Hence use of this compound during maintenance of shrimp population in ponds, tanks or natural reservoirs might decrease the interactions between PmRab7 and the viral protein.

Reduction in the interactions between the shrimp receptor protein PmRab7 and Viral envelop protein VP28 can further block one of the major route of entry of the virus into the shrimps. Further wet lab experimental validation as mentioned above can pave way for the generation of potential drug that can inhibit the entry of WSSV into the shrimp by this route, which in turn, can reduce the WSSV occurrence in the shrimp Penaeus monodon.

## Figures and Tables

**Table 1:** Structural details of the selected Phytochemicals and GDP

Chemical name	PubChem CID	Molecular Formula
Kaempferol	5280863	C15H10O6
Luteolin	5280445	C15H10O6
Quercetin	5280343	C15H10O7
Baicalein	5281605	C15H10O5
Guanosine 5'-diphosphate	8977	C10H15N5O11P2

**Table 2:** The predicted Drug likeliness of the selected ligands

S. No.	Name	Molecular Weight	Acceptors	Donors	ALogP
1	Kaempferol	286.23	6	4	1.9
2	Luteolin	286.23	6	4	1.4
3	Quercetin	302.23	7	5	1.5
4	Baicalein	270.23	5	3	1.7
5	GDP	443.2	12	7	-5.4

**Table 3:** Docking energies of the selected Plant derived flavonoids against PmRab7

S.No	Ligands	Cdocker energy(Kcal/mol)
1	Quercetin	-51.87
2	Luteolin	-48.76
3	Kaempferol	-46.41
4	Baicalein	-44.05
5	GDP	-59.57

**Table 4:** The intermolecular interactions of PmRab 7 with Quercetin and GDP

Name	PmRab 7 Residues Involved	Hydrogen Bond	Charge Interaction	Hydrophobic Bond
Quercetin	G18, V19, G20, K21, Q67 & K126	4	2	
GDP	G18, V19, G20, K21, T22, S23, Q36, Q67 & K126	9	1	1
